# Domino Hepatocyte Transplantation: A Therapeutic Alternative for the Treatment of Acute Liver Failure

**DOI:** 10.1155/2018/2593745

**Published:** 2018-07-02

**Authors:** Liana Monteiro da Fonseca Cardoso, Lucio Filgueiras Pacheco Moreira, Marcelo Alves Pinto, Andrea Henriques-Pons, Luiz Anastácio Alves

**Affiliations:** ^1^Laboratório de Comunicação Celular, Instituto Oswaldo Cruz, Fundação Oswaldo Cruz, Rio de Janeiro, Brazil; ^2^Centro Estadual de Transplantes, Rio de Janeiro, Brazil; ^3^Laboratório de Desenvolvimento Tecnológico em Virologia Instituto Oswaldo Cruz, Fundação Oswaldo Cruz, Rio de Janeiro, Brazil; ^4^Laboratório de Inovações em Terapias, Ensino e Bioprodutos, Instituto Oswaldo Cruz, Fundação Oswaldo Cruz, Rio de Janeiro, Brazil

## Abstract

**Background and Aims:**

Acute liver failure (ALF) is a severe syndrome with an elevated mortality rate, ranging from 40 to 80 %. Currently, liver transplantation is the only definitive treatment for these patients and new therapies aiming to treat ALF include artificial organs implant and stem cells therapy, for example. However, a major limitation of liver donors exists. Living donor liver transplantation (LDL﻿T), split liver transplantation (SLT), and domino liver transplantation (DLT) are some of the available alternatives to treat ALF patients, but these do not reduce the number of patients on waiting lists. Herein, we discuss domino hepatocyte transplantation (DHT) using livers that would not meet transplantation criteria.

**Methods:**

We conducted a literature search on PubMed/Medline using acute liver failure, liver transplantation, hepatocyte transplantation, and domino liver transplantation as key words.

**Results:**

New sources of biochemically functional hepatocytes and therapeutic treatments, in parallel to organ transplantation, may improve liver injury recovery and decrease mortality rates. Moreover, the literature reports hepatocyte transplantation as a therapeutic alternative for organ shortage. However, a major challenge remains for a wide clinical application of hepatocytes therapy, i.e., the availability of sufficient amounts of cells for transplantation. Ideally, hepatocytes isolated from livers rejected for transplantation may be a promising alternative for this problem.

**Conclusion:**

Our review suggests that DHT may be an excellent strategy to increase cell supplies for hepatocyte transplantation.

## 1. Acute Liver Failure

Acute liver failure (ALF) is a complex clinical syndrome with rapid deterioration of liver function, generally without preexisting diseases [1]. This condition presents a high mortality rate, of up to 80% in some reports [2], and is usually associated with coagulation disorders and hepatic encephalopathy. ALF can affect other organs, such as brain, kidneys, lungs, bone marrow and the circulatory system, and immune system [3, 4]. Therefore, metabolic acidosis, coagulation disorders, and hepatic encephalopathy are quickly established. Intracranial hypertension, the major cause of mortality in ALF patients, is secondary to cerebral edema, which is the center of the process responsible for hepatic encephalopathy. Many etiologies are involved, including viral hepatitis (hepatitis viruses A, B, and E), drug-induced hepatitis, exogenous intoxication, vascular, ischemic or Budd-Chiari syndrome neoplastic infiltration (lymphoma), and fulminant septicemia [5–7].

Although life-support new therapies are available in Intensive Therapy Centers, mortality remains high. Thus, ALF patients remain largely with no effective therapeutic treatments, including organ transplantation, which is the standard therapy for acute stages of liver disease and the only procedure for hepatic function substitution. Therefore, new sources of biochemically functional hepatocytes and therapeutic treatments that may improve liver injury recovery, in parallel to organ transplant indication, can decrease mortality rates [8, 9].

## 2. Transplantation

The first human liver transplant (LT) was performed by Thomas Starzl in 1963 (Denver- USA) [10] and, since then, this remains the only efficient therapy for liver function recovery. Since then many strategies have been developed worldwide, such as surgical techniques, immunosuppressive agents, organ allocation, donor selection, infection prophylaxis, and the prevention of recurrent diseases [11]. As a result, survival after LT has increased significantly in recent years. Currently, the average survival is of about 90% in the first year after transplantation and 60% at 10 years [12]. The major problem regarding LT is the reduced number of available grafts. Because of this, organ transplant cannot be offered to most patients presenting acute disorders. Consequently, a high number of patients remain on waiting lists and do not undergo surgical procedures, contributing to the high mortality rates described previously [8, 13, 14].

Many strategies are currently being developed aiming to increase organ availability for LT [39, 40]. Such strategies involve living donor liver transplantation (LDLT), split liver transplantation (SLT), and domino liver transplantation (DLT) [11, 41–45].

## 3. Alternatives to Maximize the Number of Available Grafts

### 3.1. Living Donor Liver Transplantation

The first attempt at LDLT was performed in Brazil, by Raia and colleagues in 1989 [46]. This transplant was performed in a child, who did not survive the surgery. The attempt, however, established the technical viability of the procedure [11, 47]. The first successfully performed LDLT was conducted in 1990 in Australia by Strong and colleagues, using segments of the left lateral lobe as grafts [48, 49]. LDLT using liver fragment grafts from healthy living donors has emerged as a very important option for many patients, especially pediatric patients [47, 48]. Since the first liver transplants from living donors in the late 1980s, this transplantation technique has been used as a well-established tool in modern medicine transplants. Most liver transplants are performed from living donors, especially in Asian religious grounds. Liver grafts for adult patients consist mainly of the right lateral lobe and, for pediatric recipients, mainly the left lateral lobe of the liver donor [48, 49].

Strategies for conducting LDLT in children have evolved and the increasing success rate of this surgical procedure has led to a significant decrease in pediatric patient mortality on transplant waiting lists [41]. With the success of liver transplantation from living donors using the left lateral lobe as small grafts for pediatric patients, surgeons have extended this procedure for adult recipients. This method, previously limited by the graft size, became possible after performing the first LDLT using the right lateral lobe. The first LDLT for adult recipients was performed in Japan, in 1996 and, since then, liver grafting using the right lateral lobe has become the main type of LDLT graft [11, 49].

Living organ donors are better than brain-dead organ donors in many ways. The main advantage of living donation is that it optimizes the time for transplantation and patients with severe liver diseases are removed from waiting lists. In addition, the transplant procedure can be electively programmed, before the development of end-stage liver disease in transplant candidates, which would increase mortality risks. Preservation time is minimal in LDLT and, therefore, ischemic liver damage is significantly reduced. Living donors in general terms are healthy and the quality of the organ is usually higher. However, the most important fact is that living donor transplant increases the supply of organs for transplantation, allowing more people to be benefited [11, 41, 48].

The major disadvantage of LDLT is the risk of donor mortality and morbidity. Thus, perfectly healthy volunteer donors are exposed to possible damage related to the surgical procedure. The risk of death of a donor of a segment on the left side, or left lobe, is of approximately 0.1%, while the risk of a donor of a right lobe segment is estimated at approximately 0.4 to 0.5%. The morbidity of these procedures is significant and correlates directly with the extent of resection [48].

An LDLT is technically more complex than a whole organ transplant from a deceased donor. In the case of partial transplant grafts, there is an increase in biliary complications, as well as the incidence of small-for-size syndrome, in which the recipient does not get enough functional liver mass [50]. Many questions regarding the impact of liver regeneration and risks for the recipient are still unanswered. Liver regeneration, for example, may favor viral replication (particularly hepatitis C or tumor growth) [48, 49]. It is noteworthy that this procedure is considerably more expensive and the possibilities of surgical complications are greater than those of whole organ transplantation. It takes two highly experienced surgeon staffs, one to perform the donor procedure and the other, the recipient [49, 50]. However, in recent years, most of the problems related to the technical LDLT procedures, especially in adults, have been elucidated. As a result, morbidity and mortality associated with technical errors during this type of transplant have decreased [50].

### 3.2. Split Liver Transplant

Split liver transplant (SLT) was also developed as a strategy to increase liver graft supplies, by obtaining two grafts from a single deceased donor. This strategy is of great importance, especially for pediatric patients, also reducing mortality on waiting lists [42].

Pichlmayr and collaborators in Hannover-Germany first described this type of transplant in 1988. The first SLT procedures were unsuccessful, especially in adult recipients and this technique was abandoned in the 1990s [11]. However, with a better understanding of intrahepatic anatomy, adequate criteria for donor and recipient selection, and the introduction of the technical division of the organ* in situ*, SLT became more useful. This was possible probably due to the decreased time lapse that leads to ischemia and biliary complication among children and adult recipients. Thus, from the mid-1990s, many transplant centers around the world have began executing SLT programs [42, 51].

Usually, the liver is divided for an adult and a child and the use of grafts for the division between two adults is uncommon. The split procedure of the organ is usually as follows: the left lateral lobe of the segment is used as a graft for the pediatric patient and the right lateral lobe for the adult recipient [42].

SLT can be safely used with acceptable morbidity and mortality rates. Nesher et al., in 2011, evaluated 2.301 SLTs performed from January 1995 to December 2008. Patient survival rates at 1 and 5 years after SLT were 84% and 70%, respectively. Early or late mortality of this type of transplant for these patients was not related to expected technical problems due to the liver split, but caused by sepsis, cerebral edema, and acute and chronic rejection [51].

### 3.3. Domino Transplantation

Domino liver transplantation (DLT) is considered for patients presenting certain genetic or biochemical disorders that are currently treated by liver transplantation. Thus, patients with liver metabolic diseases receive a transplant; the explanted ill liver sometimes can be transplanted to another patient in situations whose livers remain structurally normal with preserved function and in which liver transplantation is expected to be curative [52]. DLT was originally proposed to compensate the limited availability of organs, using patients with Familial Amyloid Polyneuropathy (FAP) as donors. Hence, this procedure is currently responsible for over 16% of liver transplants performed in Portugal [44]. A DLT conducted using the liver of a FAP patient was first performed in October 1995 in Portugal, by Furtado et al., and is carried out most frequently in Portugal, Sweden, and Japan, where the disease incidence is higher [53, 54]. FAP is an autosomal dominant systemic disease characterized by a progressive sensory motor neuropathy. It is associated with vegetative dysfunction and cardiomyopathy with a transthyretin (TTR) mutation of the TTR gene and usually occurs in adults during the third decade of life. TTR mutants are responsible for destabilizing the native tetrameric structure of TTR, leading to toxic extracellular deposition of amyloid fibrils, especially in the peripheral nervous system [44, 54]. The liver produces about 90% of TTR in the body. However, although these patients suffer a genetic alteration, liver function and normal morphology are maintained, except for mutant TTR production. In this case, the basic idea behind such grafts is the fact that it takes 20-30 years for the first symptoms to appear in patients receiving FAP liver grafts by domino transplantation [44, 55, 56]. Therefore, DLT recipients may benefit more from a donor with end-stage liver disease. In this type of sequential transplantation, FAP patient livers are not feasible for these patients; however, these livers may be transplanted into one or more patients with acute liver disease on waiting lists for orthotopic transplantation. FAP patients are both donor and recipient, since they depend on liver transplants from a deceased donor [44]. Although DLT is most frequently performed in donors presenting FAP, some studies have suggested the use of grafts from patients with a range of other metabolic disorders [57–59]. In some cases, DLT can be performed by transplanting the liver from patients with various metabolic disorders into elderly recipients, whose projected survival precludes prolonged waiting on the transplant list [57]. Golbus et al. in 2017 reported a DLT case using the liver of a 14-year-old boy with homozygous familial hypercholesterolemia (FH) transplanted into a 65-year-old man with primary sclerosing cholangitis and cirrhosis [57]. Another paper reported a first case of double domino liver transplantation in a 32-year-old woman who was diagnosed with FAP and liver dysfunction [58]. In this case, auxiliary DLT was conducted using a double domino graft from patients presenting different liver metabolic diseases. Moreover, domino liver grafts from patients with other liver metabolic disorders, such as maple syrup urine disease, are possible. In this disease, an autosomal recessive deficiency of branch chain *α*-ketoacid dehydrogenase (BCKAD) is observed in all tissues, an enzyme that metabolizes branch chain amino acids (valine, leucine, and isoleucine). In normal individuals, other tissues possess this enzyme. In these circumstances, a patient may receive a normal liver or normal hepatocytes, thus being cured. Diseases (or hepatocytes isolated from this organ) could be transplanted to another patient who will normally metabolize branch chain amino acids in other tissues presenting normal BCKAD activity. Other examples are methylmalonic acidemia [62], which can be used to supply the absence of cadaveric grafts. For rare metabolic liver diseases such as Acute Intermittent Porphyria (AIP), further reports and studies are required to establish DLT indication [54].

This procedure raises ethical and surgical issues. The most important ethical principle is the need to emphasize that the recipient can develop a genetic disease from the domino donor. At first, indicated recipients were those suffering from primary carcinoma, especially hepatocellular (HCC) and rare cholangiocarcinoma or secondary hepatic malignancies. Currently, these liver grafts are also transferred to patients with alcohol-related liver cirrhosis and viruses. It is noteworthy that this therapeutic procedure should be performed in patients with a shorter life expectancy than the time required to develop the symptoms of the domino donor disease (55-60 years) [54].

The main DLT drawback is the risk of metabolic liver disease transmission. Although with a low incidence, of about 3%, early manifestations of diseases were reported in DLT recipients [11]. Nevertheless, domino liver grafts are an adequate option for some recipients who might otherwise experience long wait times for liver transplantation, such as recipients with hepatocellular carcinoma [43].

### 3.4. Hepatocyte Transplantation

Alternatives to liver transplantation include blood purification therapies such as plasmapheresis, hemodiafiltration, and bioartificial livers [63]. However, these methods are still unsatisfactory, for numerous reasons, such as high cost, development of anaphylactic reactions, and formation and deposition of immunocomplexes [63–68]. With the advent of cell therapy and tissue engineering, the treatment of several tissue injuries in degenerative pathologies and previously untreatable processes became possible (Risbud* et al.*, 2004). Thus, the concept of hepatocyte transplantation (HT) emerged, which may lead to recovery of spontaneous patient recovery, increasing patient survival rate while awaiting donation. This would be very important, in view of the limitations of artificial liver function support methods currently applied. Studies approaching cellular therapies for liver diseases using animal models highlight the notable regenerative capacity of hepatocytes* in vivo*. Therefore, the transplantation of these cells plays an important role as a therapeutic innovation, leading to major advances regarding new perspectives in the treatment of liver diseases [9, 69, 70]. Cell transplantation may be advantageous compared to surgical procedures, since this is a less invasive procedure and can be repeated in sequential transplantations, with no need for chronic immunosuppression, and transplants can be performed in more than one recipient from a single donor [24, 39, 70, 71]. Published data reported the successful application of allogeneic hepatocytes transplants for inherited metabolic liver disease (Table 1) and ALF (Table 2). The main indications for hepatocyte transplantation treatment are (1) inherited metabolic diseases, such as Crigler-Najjar disease (CND); Glycogen storage disease 1a and 1b (GSD); ornithine transcarbamylase deficiency (OTD); familial hypercholesterolemia; progressive familial intrahepatic cholestasis (PFIC) and (2) ALF [72, 73].

The aim of hepatocyte transplantation in patients presenting metabolic disorders is to restore hepatic functions without replacing the entire organ. However, the number of cells necessary for correcting a deficit is unknown. For example, in patients suffering from Crigler-Najjar syndrome, approximately 12% of the liver mass is necessary [74]. However, fewer cells can produce an effect on other congenital disorders, such as OTD or GSD1a [75]. To be successfully performed, a minimal number of cells must be used for tissue engineering by hepatocyte transplantation. As the liver corresponds to approximately 2.8% of the total body weight, the organ from a 70-kg individual weights about 1.5 kg. The minimum liver mass required for patient survival has been asserted as about 10–30% of the total organ or 200–600 g. Considering 120 million human hepatocytes/gram of tissue, a minimum of 2.5–7.5 billion cells would be required for a clinical therapeutic treatment [75]. Nevertheless, major challenges for the wide clinical application of hepatocyte therapy include availability, metabolic integrity, and sufficient amount of cells for transplantation [33, 39, 70, 71, 73].

Many hepatocytes animal sources have been proposed and investigated as alternatives to human hepatocytes, including human tumor cell lines, immortalized hepatocytes from pig livers, and hepatocytes from transgenic pigs [75]. Each of these sources presents disadvantages for clinical use, such as the risk of disease transmission, neoplastic transformation, and animal sample biocompatibility. Ideally, primary hepatocytes isolated from human livers would be the best source of liver tissue for cell therapy, given the limitations of the cell sources mentioned above. However, the development of a standardized hepatocyte isolation to ensure adequate performance, viability, and functionality is necessary, as well as adequate conservation and storage of these cells after isolation [39]. Transplanted cells are generally no longer observed after 6–9 months and it is not clear if this is due to rejection, apoptosis, or other causes. The single most important obstacle for hepatocytes transplantations is the limited availability of hepatocytes, which has encouraged more investigations on alternative cell sources [72].

### 3.5. Systemizing a New Concept: Domino Hepatocyte Transplantation (DHT)

**Figure 1:**
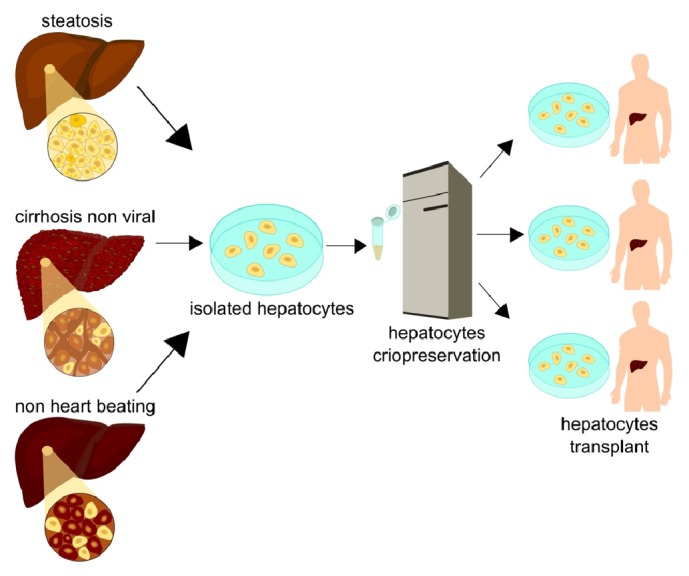
Each year a large number of livers from cadaverous donors are rejected for transplantation. The causes include a high degree of steatosis, no heart beating, and nonviral cirrhosis. These organs rejected for orthotopic transplantation have been object of studies and sources of great importance for obtaining liver cells for cell transplantation. The hepatocyte isolation with quality from these organs associated with cryopreservation technique will allow the development of a bank of liver cells that are available for the treatment of a large number of patients with ALF.

Domino transplantation is one of the strategies currently performed to supply LT grafts, mainly using unviable organs for certain individuals, such as FAP patients. In this sequential transplantation process, positive FAP grafts are functionally and morphologically normal, which enables their use as explants for patients with IHA and inherited metabolic liver diseases [44, 55, 61, 76]. This additional source of cells and strategic approach, associated with the suitable results of human hepatocyte transplantation, are considered a complementary therapeutic strategy to organ transplantation [37, 77], the domino hepatocyte transplantation (DHT) (Figure 1). Thus, unviable organs for transplantation would become sources of viable hepatocytes for transplantation. These hepatocytes could be transplanted into more than one patient presenting IHA or hepatic congenital metabolic disorders. Each year, a large number of cadaverous donor livers for transplantation are rejected. The reasons include high-grade steatosis, nonviral cirrhosis, and death due to heart failure [72, 78]. Moreover, patients with many types of metabolic liver diseases have morphologically and biochemically normal livers, except for the mutation that characterizes the metabolic disease [40, 72]. Baccarani* et al*. demonstrated that hepatocytes isolated from livers with nonviral cirrhosis, macrosteatosis, and normal livers have comparable metabolic functions when maintained in culture. Furthermore, microscopic analyses revealed normal cell morphology. In another study published by Gramignoli* et al.,* 2013, hepatocytes were isolated from explants of normal livers and livers presenting various types of inherited metabolic diseases. This group demonstrated, by using liver function assays, such as drug and ammonia metabolism and conjugation, that the cells from most metabolic diseases cases were able to perform these functions as well as, or better than, cells from normal donors. Thus, hepatocytes from a Crigler-Najjar explant could be expected to metabolize ammonia normally if transplanted to an OTD patient, for example, [40].

Hepatocyte transplantation is performed with the purpose of repopulating or replacing only a small portion of the total liver mass. Thus, the domino hepatocyte transplant could present significant advantages against the domino transplant of the whole organ. Since only a small percentage of transplanted cells present a mutation of a certain metabolic disease, this small population might not be enough for the development of the congenital disease in the recipient subject. If positive PAF receptor grafts and other inherited metabolic diseases take about 20-30 years until the onset of symptoms of the disease [44], DHT could represent an increment of this timeframe, or even the possibility of maintaining asymptomatic patients. Nevertheless, it is still too early to know the true proportion of domino recipients who will develop the disease, but a relatively large number of patients are soon to reach to develop the symptoms, 7 to 9 years after domino transplantation [43].

Cell therapy success is directly associated with the development of an efficient cryopreservation technique, capable of supplying a sufficient amount of viable and metabolically functional cells to hepatocyte cell banks for emergency transplantations or programmed transplantations for a high number of patients with diseases liver [9, 19, 27]. According to the hepatocyte infusion data displayed in Tables 1 and 2, about 10^9^ cells are required for cell therapy. Since the data are promising, especially in cases of metabolic diseases, and cryopreservation is essential for establishing cell transplant techniques, organs rejected for transplants can save the lives of patients who currently die on liver transplantation waiting lists. Such an organ cell pool may be stored in liver cell banks available for emergency or programmed DHT.

## Figures and Tables

**Table 1:** Human hepatocyte transplantation: clinical studies in patients with metabolic liver disease.

**Disease**	**Patient Age**	**Number of viable cells transplanted**	**Type of cell transplanted**	**Outcome**	**Study/Ref**
	8 years	7.5x10^9^	F/C	40% decrease bilirubin up to 6 months; OLT at 20 months	[15]
	9 years	7.5x10^9^	F	32% decrease in bilirubin for a few weeks; OLT after 5 months	[16]
	18 months	4.3x10^9^	C	40% decrease in bilirubin to 7 months; OLT at 8 months	[17]
	8 years	1.4x10^9^	F	30% decrease in bilirubin; OLT after 11 months	[18]
	1 year	2.6x10^9^	F/C	25% decrease in bilirubin at 4 months; OLT after 4 months	[19]
	9 years	6.1x10^9^	F/C	35% decrease in bilirubin at 6 months; OLT waiting list	

**Glycogen storage disease type I (GSD)**	18 years	6.0x10^9^	F/C	Improvement in glucose control, normal enzyme levels on biopsy	[20]
	47 years	2.0x10^9^	F	Better fasting time, decrease in triglycerides up to 18 months	[21]

**Infantile Refsum's disease**	4 years	2.0x10^9^	F/C	40% decrease pipecholic acid after 18 months	[22]

**Familial hyper-cholesterolemia**	Five patients between 7 and 41 years	1.1x10^9 ^	F	20% decrease in cholesterol, LDL, ApoB to 28 month;	[23]
		1.3x10^9 ^		No effect;	
		1.0x10^9 ^		6% decrease in cholesterol, LDL, ApoB to 19 months;	
		3.2x10^9 ^		Minor effect	
		1.5x10^9^		20% decrease in cholesterol up to 7 months	

**Urea cycle defects**					

OTC	5 years	1.0x10^9^	F	Decreased ammonia initially; Died 42 days later	[24]

OTC	10 hours	9.0x10^9^	F/C	Decrease ammonia; increased protein tolerance for a short period; OLT at 6 months	[25]

OTC	2 dias	1.9x10^9^	F/C	Decreased ammonia; auxiliary transplant at 6 months	[26]

OTC	14 months	2.4x10^9^	C	Decreased ammonia; increased urea; psychomotor improvement; OLT after 6 months	[27]

OTC	1 day	1.6x10^9^	F/C	Decreased ammonia; increased urea under normal diet; partial orthotopic liver transplantation (apolt) at 7 months	[28]

OTC	10 weeks 3 years	3.0x10^9^	F	Some stabilization	[29]

OTC	6 hours	0.6x10^9*∗*^	C	Decreased ammonia; increased urea; normal urinary orotic acid excretion. Death at 4 months	[30]
	9 days			Decreased ammonia; increased protein intake, urinary orotic acid normal at 6 months. OLT waiting list	

**Citrullinemia**	36 months	1.5x10^9*∗*^	C	Normal ammonia; 40% increase in urea	[30]

**Factor VII deficiency**	3 months 35 months	1.1x10^9 ^	C	70% decrease in recombinant factor VII for 6 months; OLT after 7 months	[31]
		2.2X10^9^	F/C		

**Phenylketonuria**	6 years	Two infusions for a total of 630 × 10^6^ cells	F	blood phenylalanine levels returned within the therapeutic target while the phenylalanine half-life assessed by loading tests decreased from 43 to 19 h.	[32]

**Table 2:** Human hepatocyte transplantation for patients with acute liver failure.

**Indication**	**Patient Age**	**Number of viable cells transplanted**	**Outcome**	**Study/Ref**
**Drug-induced acute liver failure**	27 years	2.8x10^7^	OLT after 10 days	[24]
	26 years	2.8x10^7^	OLT after 2 days	[33]
	32 years	1.3x10^9^	Death on day 14	[34]
	35 years	1.0x10^10^	Death on day 20	
	55 years	3.9x10^10^	Death in 6 hours	
	27 years	3.0x10^7^	OLT on day 10	[35]
	26 years	1.2x10^9^	OLT on day 2	
	21 years	9.4x10^8^	Death on day 1	
	35 years	5.4x10^9^	Death on day 18	
	35 years	3.7x10^9^	Full recovered after OLT	
	51 years	3.9x10^9^	OLT on day 3	

**Viral-induced acute liver failure**	28 years	1.9x10^7^	OLT on day 3	[24]
	28 years	1.7x10^8^	OLT on day 3	[33]
	37 years	1.2x10^8^	Death on day 5	
	43 years	7.3x10^8^	OLT on day 1	
	37 years	8.8x10^8^	Full recovered no OLT	[36]
	29 years	1.0x10^10^	Death in 18 hours	[34]
	65 years	3.0x10^10^	Death on day 52	
	4 years	3.4x10^9^	Death on day 2	[35]
	54 years	6.6x10^9^	Death on day 7	

**Idiopathic acute liver failure**	3.5 months	1.8x10^10^	OLT on day 1	[35]

**Retransplantation**	42 years 49 years	2,209 × 10^6^ hepatocytes given in 4 infusions	death on day 10 alive, liver retransplantation on day 6	[37]

**Acute-on-chronic liver failure**	7 patients with mean age, 35.7 years	(4.2 - 6.0) x 10^6^	3 patients fully recovered from liver failure, 1 survived and subsequently underwent OLT, and the remaining 3 patients died between 2.5 and 12 months hepatocyte transplantation	[38]

## Abbreviations

ALF: Acute liver failure
ASL: Arginine succinyl lyase deficiency
CND: Crigler-Najjar disease
DHT: Domino hepatocyte transplantation
DLT: Domino liver transplantation
FAP: Familial amyloid polyneuropathy
GSD: Glycogen storage disease
HCC: Hepatocellular
HT: Hepatocyte transplantation
DLT: Living donor liver transplantation
LT: Liver transplant
SLT: Split liver transplantation
OLT: Orthotopic liver transplantation
OTD: Ornithine transcarbamylase deficiency
PFIC: Progressive familial intrahepatic cholestasis
TTR: Transthyretin.

## References

[1] Scott T. R., Kronsten V. T., Hughes R. D., Shawcross D. L. (2013). Pathophysiology of cerebral oedema in acute liver failure. *World Journal of Gastroenterology*.

[2] Aleem Khan A. (2006). Journey from hepatocyte transplantation to hepatic stem cells: a novel treatment strategy for liver diseases. *Indian Journal of Medical Research*.

[3] Bernal W., Auzinger G., Dhawan A., Wendon J. (2010). Acute liver failure. *The Lancet*.

[4] Bernal W., Wendon J. (2014). Acute Liver Failure. *The New England Journal of Medicine*.

[5] Donovan J. P., Schafer D. F., Shaw B. W., Sorrell M. F. (1998). Cerebral oedema and increased intracranial pressure in chronic liver disease. *The Lancet*.

[6] Häussinger D., Kircheis G., Fischer R., Schliess F., Dahl S. V. (2000). Hepatic encephalopathy in chronic liver disease: A clinical manifestation of astrocyte swelling and low-grade cerebral edema?. *Journal of Hepatology*.

[7] Vaquero J., Chung C., Cahill M. E., Blei A. T. (2003). Pathogenesis of hepatic encephalopathy in acute liver failure. *Seminars in Liver Disease*.

[8] Lopez P. M., Martin P. (2006). Update on liver transplantation: Indications, organ allocation, and long-term care. *Mount Sinai Journal of Medicine*.

[9] Stephenne X., Najimi M., Sokal E. M. (2010). Hepatocyte cryopreservation: is it time to change the strategy?. *World Journal of Gastroenterology*.

[10] Starzl T. E., Marchioro T. L., Von Kaulla K. N., Hermann G. (1963). Homotransplantation of the Liver in Humans. *Surg Gynecol Obstet*.

[11] Song A. T. W., Avelino-Silva V. I., Pecora R. A. A., Pugliese V., D'Albuquerque L. A. C., Abdala E. (2014). Liver transplantation: Fifty years of experience. *World Journal of Gastroenterology*.

[12] Rubín A., Sánchez-Montes C., Aguilera V., Juan F. S., Ferrer I., Moya A. (2013). Long-term outcome of 'long-term liver transplant survivors'. *Transplant International*.

[13] Sagmeister M., Mullhaupt B., Kadry Z., Kullak-Ublick G. A., Clavien P. A., Renner E. L. (2002). Cost-effectiveness of cadaveric and living-donor liver transplantation. *Transplantation*.

[14] Trotter J. F., Mackenzie S., Wachs M. (2003). Comprehensive cost comparison of adult-adult right hepatic lobe living-donor liver transplantation with cadaveric transplantation. *Transplantation*.

[15] Darwish A. A., Sokal E., Stephenne X., Najimi M., de Ville de Goyet J., Reding R. (2004). Permanent access to the portal system for cellular transplantation using an implantable port device. *Liver Transplantation*.

[16] Ambrosino G., Varotto S., Strom S. C. (2005). Isolated hepatocyte transplantation for Crigler-Najjar syndrome type 1. *Cell Transplantation*.

[17] Dhawan A., Mitry R. R., Hughes R. D. (2006). Hepatocyte transplantation for liver-based metabolic disorders. *Journal of Inherited Metabolic Disease*.

[18] Allen K. J., Mifsud N. A., Williamson R., Bertolino P., Hardikar W. (2008). Cell-mediated rejection results in allograft loss after liver cell transplantation. *Liver Transplantation*.

[19] Lysy P. A., Najimi M., Stéphenne X., Bourgois A., Smets F., Sokal E. M. (2008). Liver cell transplantation for Crigler-Najjar syndrome type I: Update and perspectives. *World Journal of Gastroenterology*.

[20] Lee K.-W., Lee J.-H., Sung W. S. (2007). Hepatocyte transplantation for glycogen storage disease type Ib. *Cell Transplantation*.

[21] Muraca M., Gerunda G., Neri D. (2002). Hepatocyte transplantation as a treatment for glycogen storage disease type 1a. *The Lancet*.

[22] Sokal E. M., Smets F., Bourgois A. (2003). Hepatocyte transplantation in a 4-year-old girl with peroxisomal biogenesis disease: Technique, safety, and metabolic follow-up. *Transplantation*.

[23] Grossman M., Rader D. J., Muller D. W. M. (1995). A pilot study of ex vivo gene therapy for homozygous familial hypercholesterolaemia. *Nature Medicine*.

[24] Strom S. C., Fisher R. A., Thompson M. T. (1997). Hepatocyte transplantation as a bridge to orthotopic liver transplantation in terminal liver failure. *Transplantation*.

[25] Horslen S. P., McCowan T. C., Goertzen T. C. (2003). Isolated hepatocyte transplantation in an infant with a severe urea cycle disorder. *Pediatrics*.

[26] Mitry R. R., Dhawan A., Hughes R. D. (2004). One liver, three recipients: Segment IV from split-liver procedures as a source of hepatocytes for cell transplantation. *Transplantation*.

[27] Stéphenne X., Najimi M., Smets F., Reding R., De Ville De Goyet J., Sokal E. M. (2005). Cryopreserved liver cell transplantation controls ornithine transcarbamylase deficient patient while awaiting liver transplantation. *American Journal of Transplantation*.

[28] Puppi J., Tan N., Mitry R. R. (2008). Hepatocyte transplantation followed by auxiliary liver transplantation - A novel treatment for ornithine transcarbamylase deficiency. *American Journal of Transplantation*.

[29] Meyburg J., Hoerster F., Weitz J., Hoffmann G. F., Schmidt J. (2008). Use of the Middle Colic Vein for Liver Cell Transplantation in Infants and Small Children. *Transplantation Proceedings*.

[30] Meyburg J., Das A. M., Hoerster F. (2009). One liver for four children: First clinical series of liver cell transplantation for severe neonatal urea cycle defects. *Transplantation*.

[31] Dhawan A., Mitry R. R., Hughes R. D. (2004). Hepatocyte transplantation for inherited factor VII deficiency. *Transplantation*.

[32] Stéphenne X., Debray F. G., Smets F. (2012). Hepatocyte transplantation using the domino concept in a child with tetrabiopterin nonresponsive phenylketonuria. *Cell Transplantation*.

[33] Strom S. C., Chowdhury J. R., Fox I. J. (1999). Hepatocyte transplantation for the treatment of human disease. *Seminars in Liver Disease*.

[34] Bilir B. M., Guinette D., Karrer F. (2000). Hepatocyte transplantation in acute liver failure. *Liver Transplantation*.

[35] Fisher R. A., Strom S. C. (2006). Human hepatocyte transplantation: Worldwide results. *Transplantation*.

[36] Fisher R. A., Bu D., Thompson M. (2000). Defining hepatocellular chimerism in a liver failure patient bridged with hepatocyte infusion. *Transplantation*.

[37] Pareja E., Gomez-Lechon M. J., Cortes M., Bonora-Centelles A., Castell J. V., Mir J. (2013). Human hepatocyte transplantation in patients with hepatic failure awaiting a graft. *European Surgical Research*.

[38] Wang F., Zhou L., Ma X. (2014). Monitoring of intrasplenic hepatocyte transplantation for acute-on-chronic liver failure: A prospective five-year follow-up study. *Transplantation Proceedings*.

[39] Baccarani U., Sanna A., Cariani A. (2003). Isolation of human hepatocytes from livers rejected for liver transplantation on a national basis: Results of a 2-year experience. *Liver Transplantation*.

[40] Gramignoli R., Tahan V., Dorko K. (2013). New potential cell source for hepatocyte transplantation: Discarded livers from metabolic disease liver transplants. *Stem Cell Research*.

[41] Emre S. (2001). Living donor liver transplantation: A critical review. *Transplantation Proceedings*.

[42] Emre S., Umman V. (2011). Split liver transplantation: An overview. *Transplantation Proceedings*.

[43] Kitchens W. H. (2011). Domino liver transplantation: Indications, techniques, and outcomes. *Transplantation Reviews*.

[44] Antonini T. M., Lozeron P., Lacroix C. (2013). Reversibility of acquired amyloid polyneuropathy after liver retransplantation. *American Journal of Transplantation*.

[45] Rai R. (2013). Liver Transplantatation- an Overview. *Indian Journal of Surgery*.

[46] Raia S., Nery J. R., Mies S. (1989). Liver transplantation from live donors. *The Lancet*.

[47] Rauchfuss F., Bauschke A., Bärthel E., Scheuerlein H. (2013). Living Donor Liver Transplantation - Past and Present. *Zentralbl Chir*.

[48] Florman S., Miller C. M. (2006). Live donor liver transplantation. *Liver Transplantation*.

[49] Brown R. S. (2008). Live donors in liver transplantation. *Gastroenterology*.

[50] Lee S.-G. (2015). A complete treatment of adult living donor liver transplantation: A review of surgical technique and current challenges to expand indication of patients. *American Journal of Transplantation*.

[51] Nesher E., Island E., Tryphonopoulos P. (2011). Split liver transplantation. *Transplantation Proceedings*.

[52] Wilczek H. E., Larsson M., Yamamoto S., Ericzon B.-G. (2008). Domino liver transplantation. *Journal of Hepato-Biliary-Pancreatic Sciences*.

[53] Furtado A., Tomé L., Oliveira F. J., Furtado E., Viana J., Perdigoto R. (1997). Sequential liver transplantation. *Transplantation Proceedings*.

[54] Popescu I., Dima S. O. (2012). Domino liver transplantation: How far can we push the paradigm?. *Liver Transplantation*.

[55] Barreiros G. A. P., Galle P. R., Otto G. (2013). Familial amyloid polyneuropathy. *Digestive Diseases*.

[56] Ohdan H. (2014). Response: Domino liver transplantation as a valuable option. *Transplant International*.

[57] Golbus J. R., Farhat L., Fontana R. J., Rubenfire M. (2017). Rapidly progressive atherosclerosis after domino liver transplantation from a teenage donor with homozygous familial hypercholesterolemia. *Journal of Clinical Lipidology*.

[58] Zhu Z.-J., Wei L., Qu W. (2017). First case of cross-auxiliary double domino donor liver transplantation. *World Journal of Gastroenterology*.

[59] Schielke A., Conti F., Goumard C., Perdigao F., Calmus Y., Scatton O. (2015). Liver transplantation using grafts with rare metabolic disorders. *Digestive and Liver Disease*.

[60] Khanna A., Hart M., Nyhan W. L., Hassanein T., Panyard-Davis J., Barshop B. A. (2006). Domino liver transplantation in maple syrup urine disease. *Liver Transplantation*.

[61] Badell I. R., Hanish S. I., Hughes C. B. (2013). Domino liver transplantation in maple syrup urine disease: A case report and review of the literature. *Transplantation Proceedings*.

[62] Khanna A., Gish R., Winter S. C., Nyhan W. L., Barshop B. A. (2016). Successful Domino Liver Transplantation from a Patient with Methylmalonic Acidemia. *JIMD Reports*.

[63] Struecker B., Raschzok N., Sauer I. M. (2014). Liver support strategies: Cutting-edge technologies. *Nature Reviews Gastroenterology & Hepatology*.

[64] Abouna G. M., Boehmig H. G., Serrou B., Amemiya H., Martineau G. (1970). Long-term hepatic support by intermittent multi-species liver perfusions. *The Lancet*.

[65] Uchino J., Tsuburaya T., Kumagai F., Hase T., Hamada T. (1988). A hybrid bioartificial liver composed of multiplated hepatocyte monolayers. *ASAIO Trans*.

[66] Gerlach J. C., Encke J., Hole O., Müller C., Ryan C. J., Neuhaus P. (1994). Bioreactor for a larger scale hepatocyte in vitro perfusion. *Transplantation*.

[67] Naruse K. (2005). Artificial liver support: Future aspects. *The International Journal of Artificial Organs*.

[68] Krisper P., Stauber R. E. (2007). Technology Insight: artificial extracorporeal liver support—how does Prometheus® compare with MARS?. *Nature Clinical Practice Nephrology*.

[69] Eroglu A., Russo M. J., Bieganski R. (2000). Intracellular trehalose improves the survival of cryopreserved mammalian cells. *Nature Biotechnology*.

[70] Nussler A., Konig S., Ott M. (2006). Present status and perspectives of cell-based therapies for liver diseases. *Journal of Hepatology*.

[71] Strom S. C., Fisher R. A., Rubinstein W. S. (1997). Transplantation of human hepatocytes. *Transplantation Proceedings*.

[72] Fitzpatrick E., Mitry R. R., Dhawan A. (2009). Human hepatocyte transplantation: State of the art. *Journal of Internal Medicine*.

[73] Dhawan A., Puppi J., Hughes R. D., Mitry R. R. (2010). Human hepatocyte transplantation: current experience and future challenges. *Nature Reviews Gastroenterology & Hepatology*.

[74] Asonuma K., Gilbert J. C., Stein J. E., Takeda T., Vacanti J. P. (1992). Quantitation of transplanted hepatic mass necessary to cure the gunn rat model of hyperbilirubinemia. *Journal of Pediatric Surgery*.

[75] Vacanti J. P., Kulig K. M. (2014). Liver cell therapy and tissue engineering for transplantation. *Seminars in Pediatric Surgery*.

[76] Baccarani U., Sanna A., Cariani A. (2005). Cryopreserved human hepatocytes from cell bank: In vitro function and clinical application. *Transplantation Proceedings*.

[77] Bonora-Centelles A., Donato M. T., Lahoz A. (2010). Functional characterization of hepatocytes for cell transplantation: Customized cell preparation for each receptor. *Cell Transplantation*.

[78] Hughes R. D., Mitry R. R., Dhawan A. (2006). Isolation of hepatocytes from livers from non-heart-beating donors for cell transplantation. *Liver Transplantation*.

